# Hospital admission and emergency care attendance risk for SARS-CoV-2 delta (B.1.617.2) compared with alpha (B.1.1.7) variants of concern: a cohort study

**DOI:** 10.1016/S1473-3099(21)00475-8

**Published:** 2022-01

**Authors:** Katherine A Twohig, Tommy Nyberg, Asad Zaidi, Simon Thelwall, Mary A Sinnathamby, Shirin Aliabadi, Shaun R Seaman, Ross J Harris, Russell Hope, Jamie Lopez-Bernal, Eileen Gallagher, Andre Charlett, Daniela De Angelis, Anne M Presanis, Gavin Dabrera, COVID-19 Genomics UK (COG-UK) consortium, Cherian Koshy, Amy Ash, Emma Wise, Nathan Moore, Matilde Mori, Nick Cortes, Jessica Lynch, Stephen Kidd, Derek Fairley, Tanya Curran, James McKenna, Helen Adams, Christophe Fraser, Tanya Golubchik, David Bonsall, Mohammed Hassan-Ibrahim, Cassandra Malone, Benjamin Cogger, Michelle Wantoch, Nicola Reynolds, Ben Warne, Joshua Maksimovic, Karla Spellman, Kathryn McCluggage, Michaela John, Robert Beer, Safiah Afifi, Sian Morgan, Angela Marchbank, Anna Price, Christine Kitchen, Huw Gulliver, Ian Merrick, Joel Southgate, Martyn Guest, Robert Munn, Trudy Workman, Thomas Connor, William Fuller, Catherine Bresner, Luke Snell, Amita Patel, Themoula Charalampous, Gaia Nebbia, Rahul Batra, Jonathan Edgeworth, Samuel Robson, Angela Beckett, David Aanensen, Anthony Underwood, Corin Yeats, Khalil Abudahab, Ben Taylor, Mirko Menegazzo, Gemma Clark, Wendy Smith, Manjinder Khakh, Vicki Fleming, Michelle Lister, Hannah Howson-Wells, Louise Berry, Tim Boswell, Amelia Joseph, Iona Willingham, Carl Jones, Christopher Holmes, Paul Bird, Thomas Helmer, Karlie Fallon, Julian Tang, Veena Raviprakash, Sharon Campbell, Nicola Sheriff, Victoria Blakey, Lesley-Anne Williams, Matthew Loose, Nadine Holmes, Christopher Moore, Matthew Carlile, Victoria Wright, Fei Sang, Johnny Debebe, Francesc Coll, Adrian Signell, Gilberto Betancor, Harry Wilson, Sahar Eldirdiri, Anita Kenyon, Thomas Davis, Oliver Pybus, Louis du Plessis, Alex Zarebski, Jayna Raghwani, Moritz Kraemer, Sarah Francois, Stephen Attwood, Tetyana Vasylyeva, Marina Escalera Zamudio, Bernardo Gutierrez, M. Estee Torok, William Hamilton, Ian Goodfellow, Grant Hall, Aminu Jahun, Yasmin Chaudhry, Myra Hosmillo, Malte Pinckert, Iliana Georgana, Samuel Moses, Hannah Lowe, Luke Bedford, Jonathan Moore, Susanne Stonehouse, Chloe Fisher, Ali Awan, John BoYes, Judith Breuer, Kathryn Harris, Julianne Brown, Divya Shah, Laura Atkinson, Jack Lee, Nathaniel Storey, Flavia Flaviani, Adela Alcolea-Medina, Rebecca Williams, Gabrielle Vernet, Michael Chapman, Lisa Levett, Judith Heaney, Wendy Chatterton, Monika Pusok, Li Xu-McCrae, Darren Smith, Matthew Bashton, Gregory Young, Alison Holmes, Paul Randell, Alison Cox, Pinglawathee Madona, Frances Bolt, James Price, Siddharth Mookerjee, Manon Ragonnet-Cronin, Fabricia F. Nascimento, David Jorgensen, Igor Siveroni, Rob Johnson, Olivia Boyd, Lily Geidelberg, Erik Volz, Aileen Rowan, Graham Taylor, Katherine Smollett, Nicholas Loman, Joshua Quick, Claire McMurray, Joanne Stockton, Sam Nicholls, Will Rowe, Radoslaw Poplawski, Alan McNally, Rocio Martinez Nunez, Jenifer Mason, Trevor Robinson, Elaine O'Toole, Joanne Watts, Cassie Breen, Angela Cowell, Graciela Sluga, Nicholas Machin, Shazaad Ahmad, Ryan George, Fenella Halstead, Venkat Sivaprakasam, Wendy Hogsden, Chris Illingworth, Chris Jackson, Emma Thomson, James Shepherd, Patawee Asamaphan, Marc Niebel, Kathy Li, Rajiv Shah, Natasha Jesudason, Lily Tong, Alice Broos, Daniel Mair, Jenna Nichols, Stephen Carmichael, Kyriaki Nomikou, Elihu Aranday-Cortes, Natasha Johnson, Igor Starinskij, Ana da Silva Filipe, David Robertson, Richard Orton, Joseph Hughes, Sreenu Vattipally, Joshua Singer, Seema Nickbakhsh, Antony Hale, Louissa Macfarlane-Smith, Katherine Harper, Holli Carden, Yusri Taha, Brendan Payne, Shirelle Burton-Fanning, Sheila Waugh, Jennifer Collins, Gary Eltringham, Steven Rushton, Sarah O'Brien, Amanda Bradley, Alasdair Maclean, Guy Mollett, Rachel Blacow, Kate Templeton, Martin McHugh, Rebecca Dewar, Elizabeth Wastenge, Samir Dervisevic, Rachael Stanley, Emma Meader, Lindsay Coupland, Louise Smith, Clive Graham, Edward Barton, Debra Padgett, Garren Scott, Emma Swindells, Jane Greenaway, Andrew Nelson, Clare McCann, Wen Yew, Monique Andersson, Timothy Peto, Anita Justice, David Eyre, Derrick Crook, Tim Sloan, Nichola Duckworth, Sarah Walsh, Anoop Chauhan, Sharon Glaysher, Kelly Bicknell, Sarah Wyllie, Scott Elliott, Allyson Lloyd, Robert Impey, Nick Levene, Lynn Monaghan, Declan Bradley, Tim Wyatt, Elias Allara, Clare Pearson, Husam Osman, Andrew Bosworth, Esther Robinson, Peter Muir, Ian Vipond, Richard Hopes, Hannah Pymont, Stephanie Hutchings, Martin Curran, Surendra Parmar, Angie Lackenby, Tamyo Mbisa, Steven Platt, Shahjahan Miah, David Bibby, Carmen Manso, Jonathan Hubb, Meera Chand, Gavin Dabrera, Mary Ramsay, Daniel Bradshaw, Alicia Thornton, Richard Myers, Ulf Schaefer, Natalie Groves, Eileen Gallagher, David Lee, David Williams, Nicholas Ellaby, Ian Harrison, Hassan Hartman, Nikos Manesis, Vineet Patel, Chloe Bishop, Vicki Chalker, Juan Ledesma, Katherine Twohig, Matthew Holden, Sharif Shaaban, Alec Birchley, Alexander Adams, Alisha Davies, Amy Gaskin, Amy Plimmer, Bree Gatica-Wilcox, Caoimhe McKerr, Catherine Moore, Chris Williams, David Heyburn, Elen De Lacy, Ember Hilvers, Fatima Downing, Giri Shankar, Hannah Jones, Hibo Asad, Jason Coombes, Joanne Watkins, Johnathan Evans, Laia Fina, Laura Gifford, Lauren Gilbert, Lee Graham, Malorie Perry, Mari Morgan, Matthew Bull, Michelle Cronin, Nicole Pacchiarini, Noel Craine, Rachel Jones, Robin Howe, Sally Corden, Sara Rey, Sara Kumziene-SummerhaYes, Sarah Taylor, Simon Cottrell, Sophie Jones, Sue Edwards, Justin O'Grady, Andrew Page, Alison Mather, David Baker, Steven Rudder, Alp Aydin, Gemma Kay, Alexander Trotter, Nabil-Fareed Alikhan, Leonardo de Oliveira Martins, Thanh Le-Viet, Lizzie Meadows, Anna Casey, Liz Ratcliffe, David Simpson, Zoltan Molnar, Thomas Thompson, Erwan Acheson, Jane Masoli, Bridget Knight, Sian Ellard, Cressida Auckland, Christopher Jones, Tabitha Mahungu, Dianne Irish-Tavares, Tanzina Haque, Jennifer Hart, Eric Witele, Melisa Fenton, Ashok Dadrah, Amanda Symmonds, Tranprit Saluja, Yann Bourgeois, Garry Scarlett, Katie Loveson, Salman Goudarzi, Christopher Fearn, Kate Cook, Hannah Dent, Hannah Paul, David Partridge, Mohammad Raza, Cariad Evans, Kate Johnson, Steven Liggett, Paul Baker, Stephen Bonner, Sarah Essex, Ronan Lyons, Kordo Saeed, Adhyana Mahanama, Buddhini Samaraweera, Siona Silveira, Emanuela Pelosi, Eleri Wilson-Davies, Rachel Williams, Mark Kristiansen, Sunando Roy, Charlotte Williams, Marius Cotic, Nadua Bayzid, Adam Westhorpe, John Hartley, Riaz Jannoo, Helen Lowe, Angeliki Karamani, Leah Ensell, Jacqui Prieto, Sarah Jeremiah, Dimitris Grammatopoulos, Sarojini Pandey, Lisa Berry, Katie Jones, Alex Richter, Andrew Beggs, Angus Best, Benita Percival, Jeremy Mirza, Oliver Megram, Megan Mayhew, Liam Crawford, Fiona Ashcroft, Emma Moles-Garcia, Nicola Cumley, Colin Smith, Giselda Bucca, Andrew Hesketh, Beth Blane, Sophia Girgis, Danielle Leek, Sushmita Sridhar, Sally Forrest, Claire Cormie, Harmeet Gill, Joana Dias, Ellen Higginson, Mailis Maes, Jamie Young, Leanne Kermack, Ravi Gupta, Catherine Ludden, Sharon Peacock, Sophie Palmer, Carol Churcher, Nazreen Hadjirin, Alessandro Carabelli, Ellena Brooks, Kim Smith, Katerina Galai, Georgina McManus, Chris Ruis, Rose Davidson, Andrew Rambaut, Thomas Williams, Carlos Balcazar, Michael Gallagher, Áine O'Toole, Stefan Rooke, Verity Hill, Kathleen Williamson, Thomas Stanton, Stephen Michell, Claire Bewshea, Ben Temperton, Michelle Michelsen, Joanna Warwick-Dugdale, Robin Manley, Audrey Farbos, James Harrison, Christine Sambles, David Studholme, Aaron Jeffries, Leigh Jackson, Alistair Darby, Julian Hiscox, Steve Paterson, Miren Iturriza-Gomara, Kathryn Jackson, Anita Lucaci, Edith Vamos, Margaret Hughes, Lucille Rainbow, Richard Eccles, Charlotte Nelson, Mark Whitehead, Lance Turtle, Sam Haldenby, Richard Gregory, Matthew Gemmell, Claudia Wierzbicki, Hermione Webster, Thushan de Silva, Nikki Smith, Adrienn Angyal, Benjamin Lindsey, Danielle Groves, Luke Green, Dennis Wang, Timothy Freeman, Matthew Parker, Alexander Keeley, Paul Parsons, Rachel Tucker, Rebecca Brown, Matthew Wyles, Max Whiteley, Peijun Zhang, Marta Gallis, Stavroula Louka, Chrystala Constantinidou, Meera Unnikrishnan, Sascha Ott, Jeffrey Cheng, Hannah Bridgewater, Lucy Frost, Grace Taylor-Joyce, Richard Stark, Laura Baxter, Mohammad Alam, Paul Brown, Dinesh Aggarwal, Alberto Cerda, Tammy Merrill, Rebekah Wilson, Patrick McClure, Joseph Chappell, Theocharis Tsoleridis, Jonathan Ball, David Buck, John Todd, Angie Green, Amy Trebes, George MacIntyre-Cockett, Mariateresa de Cesare, Alex Alderton, Roberto Amato, Cristina Ariani, Mathew Beale, Charlotte Beaver, Katherine Bellis, Emma Betteridge, James Bonfield, John Danesh, Matthew Dorman, Eleanor Drury, Ben Farr, Luke Foulser, Sonia Goncalves, Scott Goodwin, Marina Gourtovaia, Ewan Harrison, David Jackson, Dorota Jamrozy, Ian Johnston, Leanne Kane, Sally Kay, Jon-Paul Keatley, Dominic Kwiatkowski, Cordelia Langford, Mara Lawniczak, Laura Letchford, Rich Livett, Stephanie Lo, Inigo Martincorena, Samantha McGuigan, Rachel Nelson, Steve Palmer, Naomi Park, Minal Patel, Liam Prestwood, Christoph Puethe, Michael Quail, Shavanthi Rajatileka, Carol Scott, Lesley Shirley, John Sillitoe, Michael Spencer Chapman, Scott Thurston, Gerry Tonkin-Hill, Danni Weldon, Diana Rajan, Iraad Bronner, Louise Aigrain, Nicholas Redshaw, Stefanie Lensing, Robert Davies, Andrew Whitwham, Jennifier Liddle, Kevin Lewis, Jaime Tovar-Corona, Steven Leonard, Jillian Durham, Andrew Bassett, Shane McCarthy, Robin Moll, Keith James, Karen Oliver, Alex Makunin, Jeff Barrett, Rory Gunson

**Affiliations:** a COVID-19 National Epidemiology Cell, Public Health England, London, UK; b Statistics, Modelling and Economics Department, Public Health England, London, UK; c Immunisation and Countermeasures Division, Public Health England, London, UK; d COVID-19 Genomic Analysis Cell, Public Health England, London, UK; e Joint Modelling Team, Public Health England, London, UK; f MRC Biostatistics Unit, University of Cambridge, Cambridge, UK

## Abstract

**Background:**

The SARS-CoV-2 delta (B.1.617.2) variant was first detected in England in March, 2021. It has since rapidly become the predominant lineage, owing to high transmissibility. It is suspected that the delta variant is associated with more severe disease than the previously dominant alpha (B.1.1.7) variant. We aimed to characterise the severity of the delta variant compared with the alpha variant by determining the relative risk of hospital attendance outcomes.

**Methods:**

This cohort study was done among all patients with COVID-19 in England between March 29 and May 23, 2021, who were identified as being infected with either the alpha or delta SARS-CoV-2 variant through whole-genome sequencing. Individual-level data on these patients were linked to routine health-care datasets on vaccination, emergency care attendance, hospital admission, and mortality (data from Public Health England's Second Generation Surveillance System and COVID-19-associated deaths dataset; the National Immunisation Management System; and NHS Digital Secondary Uses Services and Emergency Care Data Set). The risk for hospital admission and emergency care attendance were compared between patients with sequencing-confirmed delta and alpha variants for the whole cohort and by vaccination status subgroups. Stratified Cox regression was used to adjust for age, sex, ethnicity, deprivation, recent international travel, area of residence, calendar week, and vaccination status.

**Findings:**

Individual-level data on 43 338 COVID-19-positive patients (8682 with the delta variant, 34 656 with the alpha variant; median age 31 years [IQR 17–43]) were included in our analysis. 196 (2·3%) patients with the delta variant versus 764 (2·2%) patients with the alpha variant were admitted to hospital within 14 days after the specimen was taken (adjusted hazard ratio [HR] 2·26 [95% CI 1·32–3·89]). 498 (5·7%) patients with the delta variant versus 1448 (4·2%) patients with the alpha variant were admitted to hospital or attended emergency care within 14 days (adjusted HR 1·45 [1·08–1·95]). Most patients were unvaccinated (32 078 [74·0%] across both groups). The HRs for vaccinated patients with the delta variant versus the alpha variant (adjusted HR for hospital admission 1·94 [95% CI 0·47–8·05] and for hospital admission or emergency care attendance 1·58 [0·69–3·61]) were similar to the HRs for unvaccinated patients (2·32 [1·29–4·16] and 1·43 [1·04–1·97]; p=0·82 for both) but the precision for the vaccinated subgroup was low.

**Interpretation:**

This large national study found a higher hospital admission or emergency care attendance risk for patients with COVID-19 infected with the delta variant compared with the alpha variant. Results suggest that outbreaks of the delta variant in unvaccinated populations might lead to a greater burden on health-care services than the alpha variant.

**Funding:**

Medical Research Council; UK Research and Innovation; Department of Health and Social Care; and National Institute for Health Research.

## Introduction

As SARS-CoV-2 evolves and new variants emerge worldwide, sustained monitoring and rapid assessment of genetic changes are required to inform the public health response and health-care management of COVID-19. WHO has outlined three key criteria to designate variants of concern (VOCs) in relation to global public health: increased transmissibility, increase in virulence or change in clinical disease presentation, and decrease in effectiveness of public health and social measures or available diagnostics, vaccines, and therapeutics.^1^

One of the first VOCs, alpha (B.1.1.7), was initially detected in England in November, 2020. Alpha had increased transmissibility compared with the previous wildtype lineage,^2^, ^3^ and became the predominant lineage accounting for 95% of cases in England by early February, 2021.^4^ This variant has been identified in 154 countries and was until recently the most prevalent lineage in Europe and North America.^5^

**Research in context**

**Evidence before this study**

We did a literature review to identify all publications on the severity of the SARS-CoV-2 delta variant (B.1.617.2). We searched PubMed on June 18, 2021, using the query: “((SARS-CoV-2) OR (COVID-19) OR (coronavirus disease 2019)) AND ((B.1.617.2) OR (Delta) OR (VOC-21APR-02)) AND ((severity) OR (hospitalisation) OR (hospital) OR (emergency care) OR (mortality) OR (lethality) OR (death))”. The search was restricted to articles published from Dec 1, 2020, with no language restrictions. Only one relevant publication was found. Based on record linkage of data on 7723 delta and 11 820 alpha variant COVID-19 cases between April 1 and June 6, 2021, with routine health-care data, the EAVE II study in Scotland reported a higher risk of hospital admission within 14 days for patients with the delta variant compared with the alpha variant (hazard ratio [HR] 1·85 [95% CI 1·39–2·47). The patients had been tested through PCR tests and variant status was determined based on S-gene positivity, a proxy test for SARS-CoV-2 variant.

**Added value of this study**

This study included data on 8682 patients with the delta variant and 34 656 patients with the alpha variant, confirmed by whole-genome-sequencing. Hence, to our knowledge, it is the largest study to date to report on hospitalisation risk for the delta variant compared with the alpha variant, and the first to do so based on sequencing-confirmed variants. The HR of hospital admission within 14 days was 2·26 (95% CI 1·32–3·89) after stratification and regression adjustment for confounders. We also believe this study is the first to estimate a risk for emergency care attendance or hospital admission within 14 days; the adjusted HR was 1·45 (1·08–1·95).

**Implications of all the available evidence**

The evidence from these two studies in Scotland and England consistently suggest that patients with COVID-19 who are infected with the delta variant have approximately two times the risk of hospital admission compared with patients with the alpha variant. These findings should be considered for resource and policy planning in secondary care, particularly in areas where the delta variant is increasing and is likely to become the dominant circulating SARS-CoV-2 variant.

The B.1.617 lineage was first reported in India in December, 2020.^6^, ^7^ Following previous waves of COVID-19, the number of confirmed cases and test positivity in India rapidly increased, with the latter reaching 30% by the end of April, 2021.^8^ In Delhi, this coincided with the B.1.617 lineages overtaking the alpha lineage, accounting for 60% of all sequenced samples. During this increase, the sub-lineage delta (B.1.617.2) also increased to approximately 80% of B.1.617 cases.^8^

The delta variant was first detected in England in March, 2021, and was designated as a VOC on May 6, 2021.^9^ The proportion of COVID-19 cases in England caused by the delta variant has rapidly increased, reaching more than 50% of sequenced isolates by May 25, 2021.^10^ Studies in India have estimated that the delta variant could be up to 50% more transmissible than the alpha variant.^8^ In England, the secondary attack rate for the delta variant was found to be nearly 3%, compared with less than 2% for the alpha variant.^10^ In addition, there is evidence of modest reduction in vaccine effectiveness against infection with the delta variant.^11^ However, among patients infected with the delta variant, previous vaccination has been reported to reduce the risk of hospital admission.^12^

To inform the public health response to the delta variant, we did two analyses. First, we characterised the severity of the delta variant compared with the alpha variant by determining the relative risk of hospital attendance or admission following infection using a stratified analysis. Second, we assessed whether associations with hospital attendance outcomes were modified by vaccination.

## Methods

### Data sources and definitions

This cohort study was done in England among individuals with laboratory confirmed COVID-19. COVID-19 is a notifiable disease and Public Health England collects data on all positive cases in England held within the Second Generation Surveillance System (SGSS).^13^, ^14^ Individual-level data on patients with laboratory-confirmed COVID-19 with first positive specimen dates between March 29 and May 23, 2021, were linked with sequencing data uploaded to the Cloud Infrastructure for Big Data Microbial Bioinformatics database.^15^ Sampling for whole-genome sequencing mainly includes geographic-weighted population-level sampling of community cases, but can be supplemented by targeted selection such as recent international travellers, care homes, or National Health Service (NHS) diagnostic laboratories.^16^ Variant classification was assigned on the basis of lineage definitions from Public Health England.^17^ Patients with whole-genome-sequencing-confirmed alpha and delta variants were deterministically linked with data on vaccination,^18^ hospital care,^19^, ^20^ and mortality using NHS number.^21^ A full description of the data sources is in the appendix (p 1).

Potential cases of re-infection were removed to avoid misallocation of variants to different episodes of care by excluding observations for which the sequenced specimen collection date was more than 14 days after the specimen collection date of the individual's first recorded positive test. Observations without an NHS number could not be linked to health-care datasets and were excluded.

The surveillance activities within which this study was conducted are part of Public Health England's responsibility to monitor COVID-19 during the current pandemic. Public Health England has legal permission, provided by Regulation 3 of The Health Service (Control of Patient Information) Regulations 2002 to process confidential patient information under Sections 3(i) a–c, 3(i) d(i and ii), and 3(iii) as part of its outbreak response activities. This study falls within the research activities approved by the Public Health England Research Ethics and Governance of Public Health Practice Group.

### Hospital attendance categorisation

Hospital care data from the Emergency Care Data Set (ECDS) and Secondary Uses Service (SUS) were linked to data for patients with confirmed COVID-19 on June 7, 2021, thereby including data submitted by NHS Trusts up to June 5, 2021. Two outcomes of hospital attendance were defined: (1) hospital admission only, and (2) attendance to emergency care or hospital admission.

Due to a lag between an individual's hospital admission and submission of corresponding SUS data (up to 8 weeks), the definition of hospital admission was determined using a combination of ECDS and SUS variables, some of which exist in only one data source. Where ECDS data were available, hospital admissions were classified as COVID-19 related if a patient presented to emergency care between 1 and 14 days after the patient's first SARS-CoV-2-positive specimen date, there was no International Classification of Disease version 10 (ICD10) code indicating that the attendance was injury related, and the discharge status indicated transfer or admission.

Where SUS data were available, hospital admissions were defined using two sets of criteria. The first set of criteria defined if the hospital visit was related to COVID-19 infection and the second evaluated whether the hospital visit qualified as an admission. All hospital visits for which the attendance date was between 1 and 14 days after the first positive specimen date were considered COVID-19-related. If the admission date was the same as the specimen date, the visit was considered COVID-19 related if (1) the patient's symptom onset date recorded in the laboratory system at the time of test was reported between 1–7 days before the specimen was taken, or (2) if hospital records included ICD10 codes relevant to COVID-19 and the patient died in hospital. These criteria add the flexibility of including records with evidence of onset preceding hospital attendance and severe COVID-19 related outcomes, without including coincidental hospitalisations among infected individuals. Admissions were defined as those where the interval between admission and discharge was more than 0 days; or if the interval between admission and discharge was 0 days and either the hospital record included ICD10 codes relevant to COVID-19 symptoms, or the patient died in hospital, or both.

Attendances to emergency care were included in the second hospital attendance outcome category. A patient was defined as having a COVID-19-related emergency care attendance if ECDS data indicated presentation to emergency care between 1 and 14 days after the patient's first SARS-CoV-2-positive specimen date, there was no ICD10 code indicating that the attendance was injury-related, and discharge details did not indicate transfer or admission.

Unless meeting the criteria described in this section, individuals who first tested positive on the same date as their hospital admission or attendance date were excluded to reduce bias of routine testing at admission for non-COVID-19 related attendances.

### Covariates and confounders

Age, sex, and area of residence were extracted from SGSS for patients with COVID-19. National-level Index of Multiple Deprivation (IMD) quintile groups were matched to the patient's lower super output area of residence. IMD is an area-level measure of relative socioeconomic deprivation. Ethnicity was determined from linkage to NHS England's Hospital Episodes Statistics data and through self-reported ethnicity at the COVID-19 test request.

Recent travel was defined as a record of travel outside of the UK within 14 days before the patient's positive COVID-19 test. This indicator was derived from five data sources: public health passenger locator forms, contact tracing of patients done by Public Health England and NHS Test and Trace, travel reported in the COVID-19 test request form, records from the International Arrival COVID-19 testing programme, and additional questionnaires completed through telephone interview for patients for whom no other travel information was available.

Confounder sets were chosen for either stratification or regression adjustment on the basis of the expected strength of the association with exposure or outcomes. The initial outbreaks of the delta variant were localised to northern England and observed in south Asian ethnic groups, and increasing prevalence of the delta variant coincided with the expansion of the COVID-19 vaccination programme to younger age groups.^9^, ^22^ Therefore, the set of most likely confounders included age (10-year age bands),^23^ ethnicity (White; Asian; Black; and mixed, other, or unknown),^24^ calendar week of specimen, area of residence (lower tier local authority [LTLA]: 314 areas), and vaccination status.^11^

Additional potential confounders included sex and socioeconomic deprivation (IMD quintiles) due to association with hospitalisation risk,^23^, ^24^ and international travel within 14 days of positive test, which was more common for patients with the delta variant when its incidence first began to increase in England.^9^ There was no a-priori expectation that these variables would strongly confound the associations between variant and outcomes so they were considered for regression adjustment rather than stratification.

### Statistical analysis

Patients were followed up for a maximum of 14 days from their earliest COVID-19-positive specimen until the hospital admission or emergency care attendance date. Patients were censored at the date of death if this occurred without a previous hospital attendance event within the 14-day period.

In the primary analysis, stratified Cox regression was used to estimate hazard ratios (HRs) of the hospitalisation outcomes (hospital admission or emergency care attendance) for patients with the delta variant compared with patients with the alpha variant. Strata were created by intersecting the likely confounders. Additional potential confounders were included using main effects. Linear main effects terms for age and calendar date were used to adjust for residual confounding after stratification.

In the secondary analysis, the HRs of the hospitalisation outcomes by variant were estimated by vaccination status. The base models were refitted with an interaction term between variant and vaccination. Due to low numbers of patients with COVID-19 who had been vaccinated, and consequently low numbers within some vaccination categories, vaccination status was grouped into two categories: unvaccinated or less than 21 days since the first vaccination dose; and 21 days or more since the first vaccination dose, with or without the second dose.

In additional analyses, the proportional hazards assumption of the Cox regression model was graphically assessed using Schoenfeld residual plots and formally tested using the Schoenfeld test. Post-evaluations of the relative magnitudes of the confounders' contribution to the adjusted HRs were done by sequentially adding the adjustment variables in the order of the percentage change in the adjusted HRs for patients with the delta variant versus the alpha variant. To assess the impact of stratification versus regression modelling on the HRs and 95% CIs, the primary model was refitted with each stratification variable instead included as a regression variable.

HRs were assessed for sensitivity to stratification by alternative region or calendar period covariates, confounding due to recent international travel or symptomatic status subgroups, or to the precise outcome definitions. Details are shown in the appendix (p 8).

Data were prepared using Stata version 15.1. Statistical analyses were done in R version 4.1.0.

### Role of the funding source

The funders of the study had no role in study design, data collection, data analysis, data interpretation, writing of the report, or in the decision to submit for publication.

## Results

Of the 49 930 sequencing-confirmed cases of alpha and delta variants in England from March 29 to May 23, 2021, 43 338 were included in our analysis (appendix p 2). 5634 records were excluded due to missing NHS numbers (4240 [10·7%] of 39 677 patients with the alpha variant and 1394 [13·6%] of 10 253 patients with the delta variant). Missing NHS number occurred more frequently among Black and Asian individuals than White individuals (1512 [15·0%] of 10 075 Asian, 291 [19·3%] of 1508 Black, and 2574 [7·7%] of 33 306 White individuals), and among international travellers (871 [29·5%] of 2952 international travellers *vs* 4762 [10·1%] of 46 977 non-travellers).

34 656 patients were infected with the alpha variant and 8682 patients had the delta variant; the proportion of weekly cases by variant changed across the study period with alpha decreasing from 7593 (99·8%) of 7606 cases in the week of March 29, 2021, to 2117 (34·8%) of 6090 cases in the week of May 17, 2021. Patients with the delta variant were younger (median age 29 years [IQR 15–41]) than patients with the alpha variant (median age 31 years [17–43]). Compared with patients with the alpha variant, a greater proportion of patients with the delta variant were from an Asian background, or lived in the north west of England or London (table 1).

**Table 1:** Observed number and proportion of cases by variant and patient characteristics

	**Overall (n=43 338)**	**Alpha variant (B.1.1.7; n=34 656)**	**Delta variant (B.1.617.2; n=8682)**
**Age, years**			
<10	3564 (8·2%)	2671 (7·7%)	893 (10·3%)
10–19	9462 (21·8%)	7373 (21·3%)	2089 (24·1%)
20–29	7636 (17·6%)	6183 (17·8%)	1453 (16·7%)
30–39	9157 (21·1%)	7364 (21·2%)	1793 (20·7%)
40–49	6885 (15·9%)	5588 (16·1%)	1297 (14·9%)
50–59	3916 (9·0%)	3196 (9·2%)	720 (8·3%)
60–69	1681 (3·9%)	1375 (4·0%)	306 (3·5%)
70–79	584 (1·3%)	495 (1·4%)	89 (1·0%)
≥80	453 (1·0%)	411 (1·2%)	42 (0·5%)
**Sex**			
Female	22 162 (51·1%)	17 913 (51·7%)	4249 (48·9%)
Male	21 176 (48·9%)	16 743 (48·3%)	4433 (51·1%)
**Ethnicity**			
White	30 152 (69·6%)	25 940 (74·8%)	4212 (48·5%)
Black	1183 (2·7%)	854 (2·5%)	329 (3·8%)
Asian	8416 (19·4%)	5130 (14·8%)	3286 (37·8%)
Mixed, other, or unknown	3587 (8·3%)	2732 (7·9%)	855 (9·8%)
**Region of residence within England**			
London	3854 (8·9%)	2601 (7·5%)	1253 (14·4%)
East midlands	5021 (11·6%)	4309 (12·4%)	712 (8·2%)
East of England	3808 (8·8%)	2771 (8·0%)	1037 (11·9%)
North east	2519 (5·8%)	2385 (6·9%)	134 (1·5%)
North west	10 561 (24·4%)	6354 (18·3%)	4207 (48·5%)
South east	2381 (5·5%)	1933 (5·6%)	448 (5·2%)
South west	723 (1·7%)	573 (1·7%)	150 (1·7%)
West midlands	4135 (9·5%)	3645 (10·5%)	490 (5·6%)
Yorkshire and Humber	10 336 (23·8%)	10 085 (29·1%)	251 (2·9%)
Index of multiple deprivation, quintile*			
1	14 480 (33·4%)	11 476 (33·1%)	3004 (34·6%)
2	9474 (21·9%)	7517 (21·7%)	1957 (22·5%)
3	7326 (16·9%)	5997 (17·3%)	1329 (15·3%)
4	6737 (15·5%)	5413 (15·6%)	1324 (15·2%)
5	5321 (12·3%)	4253 (12·3%)	1068 (12·3%)
**Calendar week of specimen in 2021**			
March 29–April 4	7606 (17·6%)	7593 (21·9%)	13 (0·1%)
April 5–April 11	5635 (13·0%)	5568 (16·1%)	67 (0·8%)
April 12–April 18	4806 (11·1%)	4673 (13·5%)	133 (1·5%)
April 19–April 25	4774 (11·0%)	4431 (12·8%)	343 (4·0%)
April 26–May 2	4690 (10·8%)	4058 (11·7%)	632 (7·3%)
May 3–May 9	4985 (11·5%)	3608 (10·4%)	1377 (15·9%)
May 10–May 16	4752 (11·0%)	2608 (7·5%)	2144 (24·7%)
May 17–May 23	6090 (14·1%)	2117 (6·1%)	3973 (45·8%)
**Vaccination status at date of specimen**			
Unvaccinated	32 078 (74·0%)	25 823 (74·5%)	6255 (72·0%)
<21 days after first vaccination dose	2632 (6·1%)	2206 (6·4%)	426 (4·9%)
≥21 days after first vaccination dose	7834 (18·1%)	6172 (17·8%)	1662 (19·1%)
≥14 days after second vaccination dose	794 (1·8%)	455 (1·3%)	339 (3·9%)
**Recent international travel within 14 days before specimen**			
No	41 435 (95·6%)	33 218 (95·9%)	8217 (94·6%)
Yes	1903 (4·4%)	1438 (4·1%)	465 (5·4%)
**Symptom status at the time of specimen**			
Asymptomatic	18 593 (42·9%)	14 934 (43·1%)	3659 (42·1%)
Symptomatic	22 091 (51·0%)	17 757 (51·2%)	4334 (49·9%)
Unknown	2654 (6·1%)	1965 (5·7%)	689 (7·9%)

Data are n (%).

* Quintiles are ranked from most deprived (quintile 1) to least deprived (quintile 5).

The results of the analysis of hospital attendance outcomes among patients with the alpha variant versus the delta variant are shown in table 2. The estimated risk for hospital admission within 14 days after the specimen was taken was higher among patients with the delta variant than the alpha variant. The estimated risk for hospital admission or emergency care within 14 days was also higher among patients with the delta variant than the alpha variant.

**Table 2:** Hospitalisation outcomes for patients with the delta variant compared with patients with the alpha variant

	**Alpha variant (B.1.1.7)**	**Delta variant (B.1.617.2)**	**HR (95% CI), delta variant *vs* alpha variant**	
			Unadjusted	Adjusted*
Hospital admission within 14 days after specimen	764/34 656 (2·2%)	196/8682 (2·3%)	1·03 (0·88–1·21)	2·26 (1·32–3·89)
Hospital admission or emergency care attendance within 14 days after specimen	1448/34 656 (4·2%)	498/8682 (5·7%)	1·39 (1·25–1·53)	1·45 (1·08–1·95)

Data are n/N (%) except where otherwise stated. HR=hazard ratio.

* Stratification for age group, ethnicity, lower-tier local authority, calendar week of specimen, vaccination status; regression adjustment for age (linear), date (linear), sex, index of multiple deprivation, and international traveller status.

Table 3 shows the HRs of the hospital attendance outcomes for patients with the delta variant versus the alpha variant by vaccination status. Among patients who were unvaccinated or had less than 21 days since the first vaccination dose, patients with the delta variant had a higher estimated risk of hospital admission and a higher risk of either hospital admission or emergency care attendance than patients with the alpha variant. In the subgroup of vaccinated patients (≥21 days after first vaccination dose, with and without a second dose), no significant difference was detected in the estimated risk for either hospital attendance outcome between patients with the delta variant and patients with the alpha variant. The risk estimates for the delta versus the alpha variant among vaccinated patients were limited by low precision and wide CIs. There were no significant interactions when comparing the HRs in the vaccinated versus unvaccinated subgroups (table 3).

**Table 3:** Hospitalisation outcomes for patients with the delta variant compared with patients with the alpha variant, by vaccination status

	Alpha variant*	Delta variant*	**Adjusted HR (95% CI)**†**, delta variant *vs* alpha variant**	**p value**‡
**Hospital admission**				
Unvaccinated or <21 days after first vaccination dose	536/28 029 (1·9%)	149/6681 (2·2%)	2·32 (1·29–4·16)	..
≥21 days after first vaccination dose with or without second vaccination dose	228/6627 (3·4%)	47/2001 (2·3%)	1·94 (0·47–8·05)	0·82
**Hospital admission or emergency care attendance**				
Unvaccinated or <21 days after first vaccination dose	1095/28 029 (3·9%)	369/6681 (5·5%)	1·43 (1·04–1·97)	..
≥21 days after first vaccination dose with or without second vaccination dose	353/6627 (5·3%)	129/2001 (6·4%)	1·58 (0·69–3·61)	0·82

Data are n/N (%) except where otherwise stated. HR=hazard ratio.

* These crude descriptive frequencies are unadjusted for age and other confounders, and so they are not directly comparable between the groups.

† Stratification for age group, ethnicity, lower-tier local authority, calendar week, vaccination status; regression adjustment for age, sex, index of multiple deprivation, specimen date, and international travel status.

‡ p values are for tests for interaction between vaccination status and variant.

The Schoenfeld residuals and test showed no significant deviation from the proportional hazards assumption (appendix p 3). The post-evaluations of the confounders showed that adjusted HRs of both categories of hospital attendance outcome (hospital admission, hospital admission or emergency care attendance) changed the most when adjusted for calendar week (83% change for hospital admission, 39% change for hospital admission or emergency care attendance; appendix p 4). When including one or all stratification variables as regression variables instead, the estimated risk for both hospital attendance outcomes were consistently greater for patients with the delta variant than for patients with the alpha variant (appendix p 7). The sensitivity analyses in which the impact on the results was assessed after adjustment for alternative region or calendar period variables, symptomatic status, analyses of subgroups, or after varying the outcome definitions are shown in the appendix (pp 8–9). The estimated risk for both categories of hospital attendance outcomes was higher for patients with the delta variant than for patients with the alpha variant in all sensitivity analyses. The differences were consistently statistically significant, except the subgroup analysis by symptom status, in which the CIs were wider, and in some instances included 1.

## Discussion

New SARS-CoV-2 infections in England are increasingly caused by the delta variant. Although the proportion of cases caused by the delta variant was 20% overall during the study period, this increased to 74% of new sequenced cases in the week starting May 31, 2021.^9^ To our knowledge, this study provides the largest whole-genome-sequencing dataset for SARS-CoV-2 in a high-income country to date, enabling the assessment of hospitalisation risk for the delta variant compared with the alpha variant using linked administrative data. The results suggest that patients with the delta variant had more than two times the risk of hospital admission compared with patients with the alpha variant. Emergency care attendance combined with hospital admission was also higher for patients with the delta variant, showing increased use of emergency care services as well as inpatient hospitalisation. Similar results were observed for the subgroup of unvaccinated patients when comparing risks of both hospital care outcomes between the two variants. In the subgroup of patients who had received at least one vaccine dose (≥21 days since their first dose), the precision was too low to determine whether the risks of the outcomes were higher or similar for patients with the delta variant compared with patients with the alpha variant. It has previously been reported that vaccination leads to a similar relative reduction in the risk of hospitalisation for patients with the delta variant or the alpha variant.^12^ This is consistent with the findings in the present study: overall, the number of hospital attendances were low in the vaccinated subgroup resulting in low-precision relative risk estimates.

This analysis is strengthened by using national, timely datasets on COVID-19 cases, hospital care episodes, and vaccinations. The individual-level data included all laboratory-confirmed COVID-19 cases, up to 98% of hospital activity, and all vaccinated individuals registered with a general practitioner in England,^14^, ^18^, ^25^ with these data updated daily. Whole-genome sequencing coverage in England increased throughout the study period: for new positive tests between April 23 and May 24, 2021, more than 60% were successfully sequenced.^9^

Compared with a matched study design, the stratified Cox regression method offers the advantage of using all potential matches rather than a fixed number of patients with the alpha variant per patient with the delta variant. Confounders such as changing demographic profiles of patients by variant or local interventions over time are accounted for. However, this method results in a loss of informative observations when stratifying by many covariates, reducing precision compared with estimates based on adjustment through regression modelling.

Administrative data have several limitations in this context. First, hospital admission data received via SUS can have a reporting delay due to monthly submission periods, which could lead to confounding. This delay and potential confounding was mitigated by both using more rapid ECDS data to identify hospital admissions via presentation to emergency care and stratification by calendar time. The confounder post-evaluation found that the HRs were most changed by adjustment for calendar week, indicating that the unadjusted estimates were indeed confounded by registration delays or other calendar-period-specific factors. Also, given regression adjustment on specific calendar date, residual confounding due to registration delays seems unlikely and is expected to affect the more recent delta cases primarily, causing a slight underestimation of the HRs. Second, there was suboptimal information on the reason for a hospital visit, preventing conclusive attribution of attendance or admission to COVID-19. However, some data flags such as injury-related attendance and ICD10 codes were used as proxies to define outcomes in the primary analysis. Nevertheless, non-COVID-19-related visits might have been included, resulting in a slight underestimate of the HRs because this misclassification is not expected to differ by variant. A strength of considering alternative outcomes is that different categories of hospital use, which could indicate levels of disease severity, have been assessed; these sensitivity analyses showed some variation in HRs but estimated risks were consistently higher for patients with the delta variant than with the alpha variant. Finally, there were no available data on comorbidities, which are known to contribute to hospitalisation risk.^24^ This study instead indirectly accounted for comorbidities using related covariates, including age, sex, ethnicity, and deprivation.^26^

Linkage was not possible for all sequenced cases due to missing NHS numbers. 5634 (11·3%) of 49 930 sequenced cases during the study period were excluded for this reason. International travellers and minority ethnic groups were overrepresented among patients with missing NHS numbers. These groups were also overrepresented in the delta variant group compared with the alpha variant group. Although there are no data to suggest that the hospital attendance or admission risk would systematically differ for the excluded individuals compared with their included peers, this cannot be ruled out.

Hospital use and admission risk might be influenced by heterogenous health-care-seeking behaviour and transmission across the variants, ethnic groups, and particularly over time and area. Changes over time in hospital admission policy might have occurred—eg, due to local hospital burden or increased use of at-home pulse oximeter monitoring.^27^ Such changes might have resulted in reduced length of stay, with shorter stays less affected by reporting delays in more recent weeks. However, stratification for calendar week and area of residence should account for such differences.

The conditions for whole-genome-sequencing selection and successful sequencing might restrict the generalisability of the study findings. Samples that test positive by PCR are most likely to be successfully sequenced if they have a low enough cycle threshold value (<30), which might be more likely in patients with a high viral load. In addition, when comparing sequenced and non-sequenced samples in the study period, there was an overrepresentation in sequenced samples from patients in younger age groups and from areas in northern England. This is likely to be due to geographic area-based increases in sequencing to understand the initial outbreaks of the delta variant. There was also a higher proportion of pillar 2 (ie, community-based) samples that were sequenced compared with samples taken through pillar 1 (public health and hospital testing and routine screening).^28^ Despite the potential that a higher proportion of delta variant samples might have been sequenced due to increased regional coverage, slightly higher ascertainment is not likely to have significantly reduced detection of the alpha variant because alpha was the most prevalent variant throughout March and April, 2021. The same sequence-quality metrics were also applied across all samples and the area-level sampling would have included a mixture of individuals with the alpha variant and individuals with the delta variant. There was no expected sample prioritisation by variant based on clinical status, particularly as most sequenced samples were from community testing.

During the study period, the incidence of the delta variant in England was increasing, and so individuals with shorter times from infection to positive test (ie, more recent infections) might be overrepresented among those who tested positive. By contrast, the incidence of the alpha variant was decreasing during the study period, and so individuals with longer times to positive test (ie, less recent infections) are likely to be overrepresented.^29^ Time from infection to testing positive among the patients in this study might be dependent on symptoms that prompt someone to be tested, because most the study population had community (pillar 2) testing, rather than routine testing in hospital or for screening (pillar 1). People who test quickly might be more likely to have earlier or more symptoms than people who test less quickly, suggesting that their disease progression might have been both faster and more severe. This differential selection of patients with potentially more severe symptoms from the delta variant and patients with less severe symptoms from the alpha variant might result in an overestimation of the HRs. However, this bias might be mitigated by the overall preferential selection of patients with low cycle threshold values (higher viral load), that might affect the alpha and delta variant groups similarly. Furthermore, the estimated HRs were similar for patients who were asymptomatic at the time the specimen was taken, for whom the time from infection to test is unlikely to reflect differences in test-seeking behaviour. To address this bias, incidence would need to be modelled jointly with severity.

The impact of the delta variant within India has been substantial. Alongside high infection incidence, major cities also experienced overwhelming hospital burden leading to shortages of supplies and life-saving equipment.^6^ However, there has been little research done to quantify the hospitalisation risk for patients with this variant. The EAVE II study is a recent, large-scale study reporting on the hospital admission risk for patients with the delta variant in Scotland.^30^ Based on record-linkage of routine health-care data, it used S-gene target detection through diagnostic tests as a proxy for delta compared with the alpha profile that includes S-gene target failure. Their results showed an adjusted HR of hospital admission of 1·85 (95% CI 1·39–2·47), which is consistent with the HR of 2·26 (1·32–3·89) estimated in this study.

Supplementary sensitivity analyses provide assurance regarding the robustness of outcomes; however, future work should include metrics based on richer but less timely data on severe COVID-19 outcomes, such as length of hospital stay, admission to intensive care, or indicators of critical illness. Further work is also needed to measure the risk of mortality due to the delta variant, as a large proportion of the cohort included in this study was still within the 28-day follow-up period when analysis was done.

To our knowledge, this study is the largest assessment of hospitalisation risk for the delta variant using cases confirmed by whole-genome sequencing, providing important foundational evidence of increased risk compared with the alpha variant. Before the emergence of the delta variant, the evidence base largely focused on the alpha variant and its higher transmissibility and severity when compared with previous wildtype strains.^2^, ^3^, ^31^, ^32^, ^33^ Further research is needed to clarify if the hospitalisation risks differ in vaccinated individuals infected with the delta variant compared with the alpha variant; however, a previous study has estimated low hospitalisation risks for vaccinated individuals after infection with either variant.^12^ Together, these two studies suggest that outbreaks of the delta variant in unvaccinated populations might lead to a higher health-care burden, particularly compared with the previous prevalent SARS-CoV-2 strains. The findings are key for resource planning and policy decisions to mitigate the impact of the delta variant in the UK, where the delta variant now dominates, and in other high-income countries where the rapid spread of the delta variant might occur.

## Data sharing

This analysis was based on routine health-care data, which cannot be made available to others by the study authors. Requests to access these non-publicly available data are handled by the Public Health England Office for Data Release.

## Declaration of interests

GD's employer, Public Health England, has received funding from GlaxoSmithKline for a research project related to seasonal influenza and antiviral treatment; this project preceded and had no relation to COVID-19, and GD had no role in and received no funding from the project. All other authors declare no competing interests.
