# Supplementary material for: Hospital admission and emergency care attendance risk for SARS-CoV-2 delta (B.1.617.2) compared with alpha (B.1.1.7) variants of concern: a cohort study
Source: Lancet Infect Dis. 2022 Jan;22(1):35–42. doi: 10.1016/S1473-3099(21)00475-8 (PMC8397301; doi:10.1016/S1473-3099(21)00475-8)
Supplement: Supplementary appendix [file mmc1.pdf]

THE LANCET
Infectious Diseases

Supplementary appendix

This appendix formed part of the original submission and has been peer reviewed. We post it as supplied by the authors.

Supplement to: Twohig KA, Nyberg T, Zaidi A, et al. Hospital admission and emergency care attendance risk for SARS-CoV-2 delta (B.1.617.2) compared with alpha (B.1.1.7) variants of concern: a cohort study. *Lancet Infect Dis* 2021; published August 27. https://doi.org/10.1016/S1473-3099(21)00475-8.

Supplementary material

1.0 Data sources

Cases data

COVID-19 is a notifiable disease and Public Health England (PHE) collects data on all positive cases of COVID-19 in England held within the Second Generation Surveillance System (SGSS).[1, 2]

Variant identification

Confirmed Alpha and Delta cases were identified from sequencing information co-ordinated by COVID-19 Genomics UK consortium COG-UK, which had passed quality checks and subsequently been uploaded to the CLIMB (Cloud Infrastructure for Big Data Microbial Bioinformatics) database.[3] Variant classification was assigned based on lineage definitions from PHE.[4]

Vaccination status

Individual-level COVID-19 vaccination data, including date of vaccination and dosage, were derived from the NHS England-maintained National Immunisation Management System (NIMS),[5] which includes COVID-19 vaccination data for all individuals in England. Vaccination status at the time of positive test was calculated based on interval between the first positive specimen date and vaccination dates.

Hospital care data

Hospital care data were obtained from hospital records from the Secondary Uses Service (SUS) and Emergency Care Dataset (ECDS) data collections collated by NHS Digital.[6, 7] SUS includes data on admissions to NHS hospitals is updated monthly. ECDS provides information on emergency care attendances, including those that result in transfer or admission, and can be updated daily depending on the Trust. Hence, combining SUS and ECDS can give a more timely and complete ascertainment of hospital attendance and admission.[8]

Deaths

Mortality data were taken from the PHE COVID-19 associated deaths dataset, which consolidates deaths reported through four sources: (i) deaths in hospitals, notified to NHS England by NHS trusts, (ii) COVID-19 associated deaths notified to PHE Health Protection Teams during outbreak management, (iii) SARS-CoV-2-positive laboratory test results linked with death reports from NHS records through the Demographic Batch Service, and (iv) Office for National Statistics death registrations where COVID-19 was mentioned on the death certificate that could be retrospectively linked to a laboratory confirmed COVID-19 test.[9]

2.0 Study inclusion flow diagram

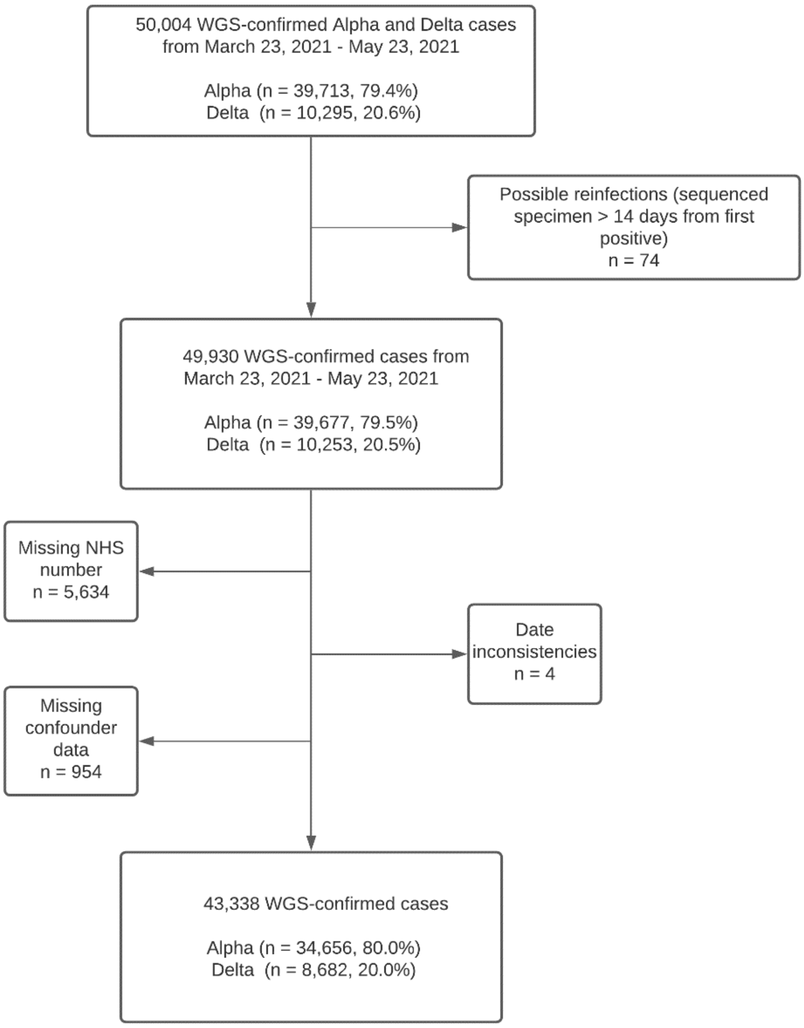

Figure S1: Inclusion flow diagram for study population

3.0 Sensitivity analyses
Proportional hazards assumption
Methods

The proportional hazards assumption of the Cox regression model was graphically assessed using plots of the scaled Schoenfeld residuals by follow-up time from the primary fully adjusted models, fitted to natural cubic spline regression curves with four degrees of freedom. If the proportional hazards assumption is true, these curves are expected to be approximately constant with no trend. The assumption was formally tested using the Schoenfeld test.

Results

The Schoenfeld residuals plots showed no clear trend for either the hospital admission (Figure S1) or the hospital admission or emergency care attendance outcome (Figure S2). Based on the Schoenfeld test, there was no significant deviation from the proportional hazards assumption for either hospital admission (p=0.241) or hospital admission or emergency care attendance (p=0.665).

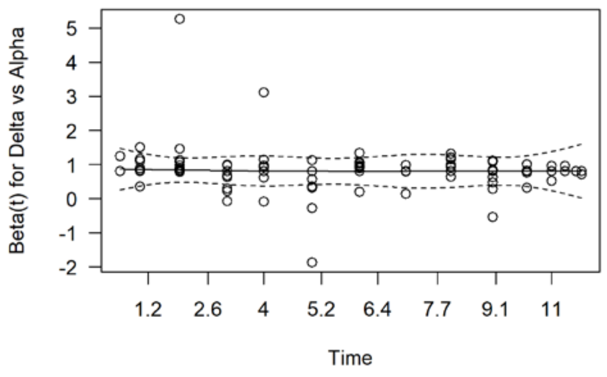

Figure S2: Schoenfeld residuals plot for the outcome hospital admission within 14 days, for Delta compared to Alpha cases.

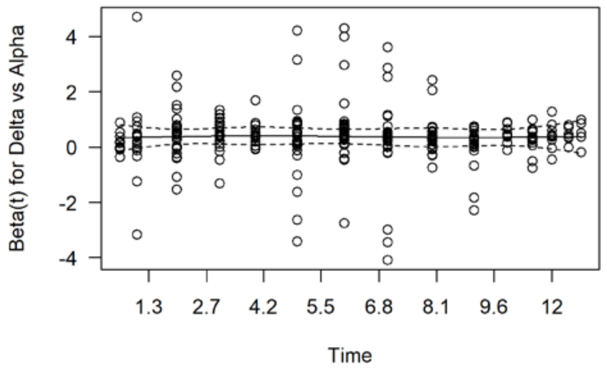

Figure S3: Schoenfeld residuals plot for the outcome hospital admission or emergency care attendance within 14 days, for Delta compared to Alpha cases.

Post-evaluation of confounder contribution to adjusted hazard ratios
Methods

Post-evaluations were performed to evaluate the relative magnitudes of the confounders' contribution to the adjusted HRs for Delta compared to Alpha cases. The adjustment models were expanded in a stepwise manner, where each considered stratification variable was added sequentially, in the order of the resulting absolute percentage change in the HRs for Delta compared to Alpha cases. After all stratification variables were added, the regression variables to adjust for the residual effects of age and calendar date were added. Next, the stepwise approach was continued with the remaining considered regression variables.

Results

For the outcome hospital admission within 14 days, the HR for Delta vs Alpha changed the most, 83%, when adjusted for calendar week. Further adjustment for ethnicity, LTLA and vaccination status changed the estimate by between 9% and 16%. Additional adjustment for age, recent international travel, IMD and sex changed the HR estimate by at most 5% (Table S1).

Similarly, for the outcome hospital admission or emergency care attendance within 14 days, the HR for Delta vs Alpha changed the most, 39%, when adjusted for calendar week. Further adjustment for ethnicity, LTLA, vaccination status and age changed the estimate by between 10% and 13%. Additional adjustment for recent international travel, IMD and sex changed the HR estimate by at most 1% (Table S2).

Step	Adjustments	HR (95% CI), Delta vs Alpha	% HR change
1	**Unadjusted**	1.03 (0.88-1.21)	
2	**Stratification for:**		
	Age group	1.18 (1.01-1.39)	15%
	Week	1.89 (1.54-2.32)	83%
	LTLA	1.09 (0.91-1.32)	6%
	Ethnicity	0.93 (0.79-1.09)	-10%
	Vaccination status	1.02 (0.87-1.20)	-1%
3	**Stratification for: Week + …**		
	Age group	1.76 (1.43-2.15)	-7%
	LTLA	2.00 (1.52-2.62)	6%
	Ethnicity	1.62 (1.31-2.01)	-14%
	Vaccination status	1.88 (1.53-2.30)	-0.5%
4	**Stratification for: Week + Ethnicity + …**		
	Age group	1.51 (1.21-1.87)	-7%
	LTLA	1.77 (1.28-2.44)	9%
	Vaccination status	1.59 (1.28-1.96)	-2%
5	**Stratification for: Week + Ethnicity + LTLA + …**		
	Age group	1.81 (1.14-2.88)	2%
	Vaccination status	2.05 (1.44-2.93)	16%
6	**Stratification for: Week + Ethnicity + LTLA + Vaccination status + …**		
	Age group	2.11 (1.24-3.59)	3%
7	**Stratification for: Week + Ethnicity + LTLA + Vaccination status + Age group; Regression adjustment for Age (linear) + Calendar date (linear)**	2.10 (1.24-3.58)	-0.5%
8	**Stratification for: Week + Ethnicity + LTLA + Vaccination status + Age group; Regression adjustment for Age (linear) + Calendar date (linear) + …**		
	Sex	2.11 (1.24-3.58)	0.5%
	IMD	2.16 (1.27-3.69)	3%

	Recent international travel	2.20 (1.28-3.78)	5%
9	**Stratification for: Week + Ethnicity + LTLA + Vaccination status + Age group; Regression adjustment for Age (linear) + Calendar date (linear) + Recent international travel + …**		
	Sex	2.21 (1.29-3.79)	0.5%
	IMD	2.26 (1.31-3.88)	3%
10	**Stratification for: Week + Ethnicity + LTLA + Vaccination status + Age group; Regression adjustment for Age (linear) + Calendar date (linear) + Recent international travel + IMD + …**		
	Sex	2.26 (1.32-3.89)	0.0%

Table S1: Post-evaluation of the contribution to the adjusted HR of hospital admission within 14 days when the adjustment variables were added sequentially

Step	Adjustments	HR (95% CI), Delta vs Alpha	% HR change
1	**Unadjusted**	1.39 (1.25-1.53)	
2	**Stratification for:**		
	Age group	1.52 (1.37-1.68)	9%
	Week	1.93 (1.69-2.20)	39%
	LTLA	1.28 (1.13-1.46)	-8%
	Ethnicity	1.25 (1.12-1.38)	-10%
	Vaccination status	1.38 (1.24-1.53)	-0.7%
3	**Stratification for: Week + …**		
	Age group	1.83 (1.60-2.09)	-5%
	LTLA	1.70 (1.42-2.03)	-12%
	Ethnicity	1.67 (1.45-1.92)	-13%
	Vaccination status	1.91 (1.67-2.18)	-1%
4	**Stratification for: Week + Ethnicity + …**		
	Age group	1.58 (1.37-1.82)	-5%
	LTLA	1.47 (1.20-1.80)	-12%
	Vaccination status	1.64 (1.43-1.89)	-2%
5	**Stratification for: Week + Ethnicity + LTLA + …**		
	Age group	1.36 (1.04-1.77)	-7%
	Vaccination status	1.62 (1.29-2.03)	10%
6	**Stratification for: Week + Ethnicity + LTLA + Vaccination status + …**		
	Age group	1.45 (1.08-1.95)	-10%
7	**Stratification for: Week + Ethnicity + LTLA + Vaccination status + Age group; Regression adjustment for Age (linear) + Calendar date (linear)**	1.41 (1.05-1.89)	-3%
8	**Stratification for: Week + Ethnicity + LTLA + Vaccination status + Age group; Regression adjustment for Age (linear) + Calendar date (linear) + …**		
	Sex	1.41 (1.05-1.90)	0.0%
	IMD	1.42 (1.06-1.91)	0.7%
	Recent international travel	1.43 (1.06-1.92)	1%
9	**Stratification for: Week + Ethnicity + LTLA + Vaccination status + Age group; Regression adjustment for Age (linear) + Calendar date (linear) + Recent international travel + …**		
	Sex	1.43 (1.06-1.93)	0.0%
	IMD	1.44 (1.07-1.94)	0.7%

10	**Stratification for: Week + Ethnicity + LTLA + Vaccination status + Age group; Regression adjustment for Age (linear) + Calendar date (linear) + Recent international travel + IMD + …**		
	Sex	1.45 (1.08-1.95)	0.7%

Table S2: Post-evaluation of the contribution to the adjusted HR of hospital admission or emergency care attendance within 14 days when the adjustment variables were added sequentially

Alternative adjustment methods
Methods

Stratification controls for confounders based on fewer assumptions than regression models, but does so at the expense of a lower effective sample size and comparatively lower power. To assess the impact of stratification versus regression modelling on the HRs and 95% CIs, the primary model was refitted with one stratification variable instead included as a regression variable, for each of the stratification variables, and also by fitting a model that used regression adjustment for all confounders. These models used a random effects term for LTLA due to the large number of categories of this covariate, and fixed effects terms for all other confounders.

Results

The primary models were informed by 30.1% of the Delta cases admitted to hospital and 35.9% of the Delta cases admitted to hospital or attending emergency care. When each one of the variables used for stratification was instead included as a regression variable, a higher proportion of the events in Delta cases informed the HR estimates. The HRs varied between 1.70 and 2.77 for hospital admission or emergency care attendance, and 1.33 and 1.67 for hospital admission or emergency care attendance, with somewhat narrower CIs compared to the HRs from the primary model. When regression adjustments were used consistently instead of stratification, by design all observations contributed to the estimates, and the HR of hospital admission was 1.66 (1.05-2.63) and the HR of hospital admission or emergency care attendance was 1.69 (1.40-2.05).

Outcome	Adjustment	Total N informative events (%)	N Delta informative events (%)	HR (95% CI), Delta vs Alpha
Hospital admission within 14 days	Primary model: Stratification for age group, ethnicity, LTLA, week, vaccination status; regression adjustment for age (linear), calendar date (linear), sex, IMD and traveller status	88 (9.2%)	59 (30.1%)	2.26 (1.32-3.89)
	Primary model as above, but using regression adjustment instead of stratifying for: Age group	218 (22.7%)	121 (61.7%)	2.04 (1.40-2.99)
	Primary model as above, but using regression adjustment instead of stratifying for: Ethnicity	167 (17.4%)	114 (58.2%)	2.77 (1.83-4.20)
	Primary model as above, but using regression adjustment (random effect) instead of stratifying for: LTLA	666 (69.4%)	189 (96.4%)	1.70 (1.12-2.59)
	Primary model as above, but using regression adjustment instead of stratifying for: Week	418 (43.5%)	148 (75.5%)	1.81 (1.35-2.42)
	Primary model as above, but using regression adjustment instead of stratifying for: Vaccination status	110 (11.5%)	68 (34.7%)	1.83 (1.13-2.95)
	Using regression adjustment for all adjustment variables	960 (100.0%)	196 (100.0%)	1.66 (1.05-2.63)
Hospital admission or emergency care attendance within 14 days	Primary model: Stratification for age, ethnicity, LTLA, week, vaccination status; regression adjustment for age (linear), calendar date (linear), sex, IMD and traveller status	280 (14.4%)	179 (35.9%)	1.45 (1.08-1.95)
	Primary model as above, but using regression adjustment instead of stratifying for: Age group	572 (29.4%)	331 (66.5%)	1.57 (1.25-1.98)
	Primary model as above, but using regression adjustment instead of stratifying for: Ethnicity	486 (25.0%)	306 (61.4%)	1.62 (1.27-2.05)
	Primary model as above, but using regression adjustment (random effect) instead of stratifying for: LTLA	1475 (75.8%)	482 (96.8%)	1.67 (1.40-1.99)
	Primary model as above, but using regression adjustment instead of stratifying for: Week	1015 (52.2%)	389 (78.1%)	1.59 (1.32-1.92)
	Primary model as above, but using regression adjustment instead of stratifying for: Vaccination status	357 (18.3%)	221 (44.4%)	1.33 (1.02-1.74)
	Using regression adjustment for all adjustment variables	1946 (100.0%)	498 (100.0%)	1.69 (1.40-2.05)

Table S3: Analysis to assess the impact of stratification versus regression modelling on the effective sample size, and HRs and 95% CIs for Delta vs Alpha cases

Alternative adjustment variables, subgroup analyses and outcome definitions
Methods

To assess the sensitivity of the HR estimates to the stratification by region or calendar period, the primary model was refitted, instead stratified by upper-tier local authority (UTLA: 150 areas), PHE Region (PHEC: 9 regions), or exact specimen date. To exclude the possibility that the estimates were driven by confounding due to recent travellers, the models were refitted after excluding this subgroup. The primary analysis did not adjust for symptomatic status due to the possibility that symptom profile may be in the causal pathway between variant and outcomes. In a sensitivity analysis, symptomatic status was instead adjusted for, or separate models were fitted to the subgroups of symptomatic or asymptomatic cases. Finally, the sensitivity of the results to the outcome definitions were assessed by varying the definition to expand or restrict the profile of events considered as outcomes.

Results

Stratifying by UTLA instead of LTLA decreased the HR estimates for hospital admission to 1.68 (1.05-2.69); and to 1.39 (1.06-1.83) for emergency care attendance or hospital admission (Table S1). Stratifying by PHEC region instead of LTLA reduced the point estimate of the HR for hospital admission, and increased the precision of the estimate (HR 1.64 (1.26-2.13)), due to larger effective sample sizes when stratifying by a broader area. Stratifying by PHEC region also increased the HR for emergency care attendance or hospital admission, to 1.61 (1.36-1.91).

Stratifying by exact calendar date instead of calendar week resulted in a similar HR for hospital admission of 2.05 (1.15-3.65) and a larger HR for emergency care attendance or hospital admission of 1.63 (1.16-2.31).

Excluding recent travellers from the analyses made little difference to the estimated HR: 2.09 (1.38-3.18) for hospital admission and 1.51 (1.17-1.95) for emergency care attendance or hospital admission.

Adjustment for symptomatic status only marginally changed the estimates: estimated HR 2.24 (1.29-3.87) for hospital admission and 1.44 (1.07-1.94) for emergency care attendance or hospital admission. Consistently, subgroup analyses by symptomatic status showed similar HR point estimates of both outcomes for symptomatic and asymptomatic cases.

When varying the outcome definitions, the estimates were similar when including injury-related events (hospital admission HR 2.26, 95% CI 1.31-3.88; emergency care attendance or hospital admission HR 1.41, 95% CI 1.05-1.89), after restricting to only consider hospital admissions with >0 days' stay in hospital (HR 2.11, 95% CI 1.08-4.10), or after restricting to only consider events 1-14 days after specimen (hospital admission HR 2.23, 95% CI 1.29-3.84; emergency care attendance or hospital admission HR 1.46, 95% CI 1.08-1.98). The HRs were somewhat lower when the outcome definitions were instead expanded to include events on day 0 that fulfilled the other criteria (hospital admission HR 1.74, 95% CI 1.09-2.76; emergency care attendance or hospital admission HR 1.41, 95% CI 1.07-1.85) or when including all events on days 0-14 regardless of the additional criteria (ICD10 codes and length of stay) used for the main outcome definitions (hospital admission HR 1.57, 95% CI 1.01-2.44; emergency care attendance or hospital admission HR 1.34, 95% CI 1.02-1.75), but the CIs consistently excluded 1.

**Sensitivity analysis**	**Hospital admission HR (95% CI), Delta vs Alpha***	**Hospital admission or emergency care attendance HR (95% CI), Delta vs Alpha***
Base model	2.26 (1.32-3.89)	1.45 (1.08-1.95)
Stratification for UTLA instead of LTLA	1.68 (1.05-2.69)	1.39 (1.06-1.83)
Stratification for PHEC region instead of LTLA	1.64 (1.26-2.13)	1.61 (1.36-1.91)
Stratification for exact calendar date instead of week †	2.05 (1.15-3.65)	1.63 (1.16-2.31)
Excluding recent travellers (n = 1903)	2.09 (1.38-3.18)	1.51 (1.17-1.95)
Adjustment for symptomatic status	2.24 (1.29-3.87)	1.44 (1.07-1.94)
Subgroup analysis: Asymptomatic cases	2.02 (0.54-7.56)	1.48 (0.76-2.91)

Subgroup analysis: Symptomatic cases	2.40 (1.06-5.46)	1.46 (0.92-2.30)
Alternative outcome definition: Including attendances due to injuries (n = 69)	2.26 (1.31-3.88)	1.41 (1.05-1.89)
Alternative outcome definition: Only considering hospitalisations with >0 days' stay in hospital	2.11 (1.08-4.10)	--
Alternative outcome definition: Only considering events 1-14 days after specimen (excluding events on day 0)	2.23 (1.29-3.84)	1.46 (1.08-1.98)
Alternative outcome definition: Including all events 0-14 days after specimen that fulfilled the criteria regarding length of stay or ICD code	1.74 (1.09-2.76)	1.41 (1.07-1.85)
Alternative outcome definition: Including all events 0-14 days after specimen regardless of length of stay or ICD code	1.57 (1.01-2.44)	1.34 (1.02-1.75)

* Adjusted HRs based on stratified Cox regression. Unless otherwise specified the models were stratified for 10-year age group, ethnicity, LTLA, week, vaccination status; and using regression adjustment for exact age, exact date, sex, IMD and traveller status.
† Stratification for exact calendar date, 10-year age group, LTLA; regression adjustment for exact age, sex, ethnicity, IMD, vaccination status and traveller status.

Table S4: Sensitivity analyses

4.0 References, Supplementary material

1. Department of Health and Social Care. Coronavirus (COVID-19) listed as a notifiable disease 2020 [updated 5 March 2020]. Available from: https://www.gov.uk/government/news/coronavirus-covid-19-listed-as-a-notifiable-disease. Accessed on: 6 June 2021
2. Clare T, Twohig KA, O'Connell A-M, Dabrera G. Timeliness and completeness of laboratory-based surveillance of COVID-19 cases in England. Public Health. 2021;194:163-6.
3. Connor TR, Loman NJ, Thompson S, Smith A, Southgate J, Poplawski R, et al. CLIMB (the Cloud Infrastructure for Microbial Bioinformatics).
4. PHE Genomics. Standardised Variant Definitions 2021 [updated 28 May 2021]. Available from: https://github.com/phe-genomics/variant_definitions/blob/main/README.md. Accessed on: June 15 2021
5. Graphnet. National Immunisation Management System 2021 [Available from: https://www.graphnethealth.com/solutions/immunisation-systems/ Accessed on: 10 June 2021
6. NHS Digital. Secondary Uses Service (SUS) 2021 [Available from: https://digital.nhs.uk/services/secondary-uses-service-sus. Accessed on: June 5 2021
7. NHS Digital. Emergency Care Data Set (ECDS) 2021 [Available from: https://digital.nhs.uk/data-and-information/data-collections-and-data-sets/data-sets/emergency-care-data-set-ecds. Accessed on: June 5 2021
8. Bhattacharya A, Collin SM, Stimson J, Thelwall S, Nsonwu O, Gerver S, et al. Healthcare-associated COVID-19 in England: a national data linkage study. medRxiv. 2021.
9. Brown AE, Heinsbroek E, Kall MM, Allen H, Beebeejaun K, Blomquist P, et al. Epidemiology of Confirmed COVID-19 Deaths in Adults, England, March–December 2020. Emerging Infectious Diseases. 2021;27(5):1468.

5.0 List of COG-UK consortium members

Funding acquisition, Leadership and supervision, Metadata curation, Project administration, Samples and logistics, Sequencing and analysis, Software and analysis tools, and Visualisation:
Samuel C Robson [13].

Funding acquisition, Leadership and supervision, Metadata curation, Project administration, Samples and logistics, Sequencing and analysis, and Software and analysis tools:
Nicholas J Loman [41] and Thomas R Connor [10, 69].

Leadership and supervision, Metadata curation, Project administration, Samples and logistics, Sequencing and analysis, Software and analysis tools, and Visualisation:
Tanya Golubchik [5].

Funding acquisition, Metadata curation, Samples and logistics, Sequencing and analysis, Software and analysis tools, and Visualisation:
Rocio T Martinez Nunez [42].

Funding acquisition, Leadership and supervision, Metadata curation, Project administration, and Samples and logistics:
Catherine Ludden [88].

Funding acquisition, Leadership and supervision, Metadata curation, Samples and logistics, and Sequencing and analysis:
Sally Corden [69].

Funding acquisition, Leadership and supervision, Project administration, Samples and logistics, and Sequencing and analysis:
Ian Johnston [99] and David Bonsall [5].

Funding acquisition, Leadership and supervision, Sequencing and analysis, Software and analysis tools, and Visualisation:
Colin P Smith [87] and Ali R Awan [28].

Funding acquisition, Samples and logistics, Sequencing and analysis, Software and analysis tools, and Visualisation:
Giselda Bucca [87].

Leadership and supervision, Metadata curation, Project administration, Samples and logistics, and Sequencing and analysis:
M. Estee Torok [22, 101].

Leadership and supervision, Metadata curation, Project administration, Samples and logistics, and Visualisation:
Kordo Saeed [81, 110] and Jacqui A Prieto [83, 109].

Leadership and supervision, Metadata curation, Project administration, Sequencing and analysis, and Software and analysis tools:
David K Jackson [99].

Metadata curation, Project administration, Samples and logistics, Sequencing and analysis, and Software and analysis tools:
William L Hamilton [22].
Metadata curation, Project administration, Samples and logistics, Sequencing and analysis, and Visualisation:
Luke B Snell [11].

Funding acquisition, Leadership and supervision, Metadata curation, and Samples and logistics:
Catherine Moore [69].

Funding acquisition, Leadership and supervision, Project administration, and Samples and logistics:
Ewan M Harrison [99, 88].

Leadership and supervision, Metadata curation, Project administration, and Samples and logistics:
Sonia Goncalves [99] and Leigh M Jackson [91].

Leadership and supervision, Metadata curation, Samples and logistics, and Sequencing and analysis:
Ian G Goodfellow [24], Derek J Fairley [3, 72], Matthew W Loose [18] and Joanne Watkins [69].

Leadership and supervision, Metadata curation, Samples and logistics, and Software and analysis tools:
Rich Livett [99].

Leadership and supervision, Metadata curation, Samples and logistics, and Visualisation:
Samuel Moses [25, 106].

Leadership and supervision, Metadata curation, Sequencing and analysis, and Software and analysis tools:
Roberto Amato [99], Sam Nicholls [41] and Matthew Bull [69].

Leadership and supervision, Project administration, Samples and logistics, and Sequencing and analysis:
Darren L Smith [37, 58, 105].

Leadership and supervision, Sequencing and analysis, Software and analysis tools, and Visualisation:
Jeff Barrett [99], David M Aanensen [14, 114].

Metadata curation, Project administration, Samples and logistics, and Sequencing and analysis:
Martin D Curran [65], Surendra Parmar [65], Dinesh Aggarwal [95, 99, 64] and James G Shepherd [48].

Metadata curation, Project administration, Sequencing and analysis, and Software and analysis tools:
Matthew D Parker [93].

Metadata curation, Samples and logistics, Sequencing and analysis, and Visualisation:
Sharon Glaysher [61].

Metadata curation, Sequencing and analysis, Software and analysis tools, and Visualisation:
Matthew Bashton [37, 58], Anthony P Underwood [14, 114], Nicole Pacchiarini [69] and Katie F Loveson [77].

Project administration, Sequencing and analysis, Software and analysis tools, and Visualisation:
Alessandro M Carabelli [88].

Funding acquisition, Leadership and supervision, and Metadata curation:
Kate E Templeton [53, 90].

Funding acquisition, Leadership and supervision, and Project administration:
Cordelia F Langford [99], John Sillitoe [99], Thushan I de Silva [93] and Dennis Wang [93].

Funding acquisition, Leadership and supervision, and Sequencing and analysis:
Dominic Kwiatkowski [99, 107], **And**rew Rambaut [90], Justin O'Grady [70, 89] and Simon Cottrell [69].

Leadership and supervision, Metadata curation, and Sequencing and analysis:
Matthew T.G. Holden [68] and Emma C Thomson [48].

Leadership and supervision, Project administration, and Samples and logistics:
Husam Osman [64, 36], Monique Andersson [59], Anoop J Chauhan [61] and Mohammed O Hassan-Ibrahim [6].

Leadership and supervision, Project administration, and Sequencing and analysis:
Mara Lawniczak [99].

Leadership and supervision, Samples and logistics, and Sequencing and analysis:

Ravi Kumar Gupta [88, 113], Alex Alderton [99], Meera Chand [66], Chrystala Constantinidou [94], Meera Unnikrishnan [94], Alistair C Darby [92], Julian A Hiscox [92] and Steve Paterson [92].

Leadership and supervision, Sequencing and analysis, and Software and analysis tools:
Inigo Martincorena [99], David L Robertson [48], Erik M Volz [39], Andrew J Page [70] and Oliver G Pybus [23].

Leadership and supervision, Sequencing and analysis, and Visualisation:
Andrew R Bassett [99].

Metadata curation, Project administration, and Samples and logistics:
Cristina V Ariani [99], Michael H Spencer Chapman [99, 88], Kathy K Li [48], Rajiv N Shah [48], Natasha G Jesudason [48] and Yusri Taha [50].

Metadata curation, Project administration, and Sequencing and analysis:
Martin P McHugh [53] and Rebecca Dewar [53].

Metadata curation, Samples and logistics, and Sequencing and analysis:
Aminu S Jahun [24], Claire McMurray [41], Sarojini Pandey [84], James P McKenna [3], Andrew Nelson [58, 105], Gregory R Young [37, 58], Clare M McCann [58, 105] and Scott Elliott [61].

Metadata curation, Samples and logistics, and Visualisation:
Hannah Lowe [25].

Metadata curation, Sequencing and analysis, and Software and analysis tools:
Ben Temperton [91], Sunando Roy [82], Anna Price [10], Sara Rey [69] and Matthew Wyles [93].

Metadata curation, Sequencing and analysis, and Visualisation:
Stefan Rooke [90] and Sharif Shaaban [68].

Project administration, Samples and logistics, Sequencing and analysis:
Mariateresa de Cesare [98].

Project administration, Samples and logistics, and Software and analysis tools:
Laura Letchford [99].

Project administration, Samples and logistics, and Visualisation:
Siona Silveira [81], Emanuela Pelosi [81] and Eleri Wilson-Davies [81].

Samples and logistics, Sequencing and analysis, and Software and analysis tools:
Myra Hosmillo [24].

Sequencing and analysis, Software and analysis tools, and Visualisation:
Áine O'Toole [90], Andrew R Hesketh [87], Richard Stark [94], Louis du Plessis [23], Chris Ruis [88], Helen Adams [4] and Yann Bourgeois [76].

Funding acquisition, and Leadership and supervision:
Stephen L Michell [91], Dimitris Grammatopoulos[84, 112], Jonathan Edgeworth [12], Judith Breuer [30, 82], John A Todd [98] and Christophe Fraser [5].

Funding acquisition, and Project administration:
David Buck [98] and Michaela John [9].

Leadership and supervision, and Metadata curation:
Gemma L Kay [70].

Leadership and supervision, and Project administration:
Steve Palmer [99], Sharon J Peacock [88, 64] and David Heyburn [69].

Leadership and supervision, and Samples and logistics:

Danni Weldon [99], Esther Robinson [64, 36], Alan McNally [41, 86], Peter Muir [64], Ian B Vipond [64], John BoYes [29], Venkat Sivaprakasam [46], Tranprit Salluja [75], Samir Dervisevic [54] and Emma J Meader [54].

Leadership and supervision, and Sequencing and analysis:
Naomi R Park [99], Karen Oliver [99], Aaron R Jeffries [91], Sascha Ott [94], Ana da Silva Filipe [48], David A Simpson [72] and Chris Williams [69].

Leadership and supervision, and Visualisation:
Jane AH Masoli [73, 91].

Metadata curation, and Samples and logistics:
Bridget A Knight [73, 91], Christopher R Jones [73, 91], Cherian Koshy [1], Amy Ash [1], Anna Casey [71], Andrew Bosworth [64, 36], Liz Ratcliffe [71], Li Xu-McCrae [36], Hannah M Pymont [64], Stephanie Hutchings [64], Lisa Berry [84], Katie Jones [84], Fenella Halstead [46], Thomas Davis [21], Christopher Holmes [16], Miren Iturriza-Gomara [92], Anita O Lucaci [92], Paul Anthony Randell [38, 104], Alison Cox [38, 104], Pinglawathee Madona [38, 104], Kathryn Ann Harris [30], Julianne Rose Brown [30], Tabitha W Mahungu [74], Dianne Irish-Tavares [74], Tanzina Haque [74], Jennifer Hart [74], Eric Witele [74], Melisa Louise Fenton [75], Steven Liggett [79], Clive Graham [56], Emma Swindells [57], Jennifer Collins [50], Gary Eltringham [50], Sharon Campbell [17], Patrick C McClure [97], Gemma Clark [15], Tim J Sloan [60], Carl Jones [15] and Jessica Lynch [2, 111].

Metadata curation, and Sequencing and analysis:
Ben Warne [8], Steven Leonard [99], Jillian Durham [99], Thomas Williams [90], Sam T Haldenby [92], Nathaniel Storey [30], Nabil-Fareed Alikhan [70], Nadine Holmes [18], Christopher Moore [18], Matthew Carlile [18], Malorie Perry [69], Noel Craine [69], Ronan A Lyons [80], Angela H Beckett [13], Salman Goudarzi [77], Christopher Fearn [77], Kate Cook [77], Hannah Dent [77] and Hannah Paul [77].

Metadata curation, and Software and analysis tools:
Robert Davies [99].

Project administration, and Samples and logistics:
Beth Blane [88], Sophia T Girgis [88], Mathew A Beale [99], Katherine L Bellis [99, 88], Matthew J Dorman [99], Eleanor Drury [99], Leanne Kane [99], Sally Kay [99], Samantha McGuigan [99], Rachel Nelson [99], Liam Prestwood [99], Shavanthi Rajatileka [99], Rahul Batra [12], Rachel J Williams [82], Mark Kristiansen [82], Angie Green [98], Anita Justice [59], Adhyana I.K Mahanama [81, 102] and Buddhini Samaraweera [81, 102].

Project administration, and Sequencing and analysis:
Nazreen F Hadjirin [88] and Joshua Quick [41].

Project administration, and Software and analysis tools:
Radoslaw Poplawski [41].

Samples and logistics, and Sequencing and analysis:
Leanne M Kermack [88], Nicola Reynolds [7], Grant Hall [24], Yasmin Chaudhry [24], Malte L Pinckert [24], Iliana Georgana [24], Robin J Moll [99], Alicia Thornton [66], Richard Myers [66], Joanne Stockton [41], Charlotte A Williams [82], Wen C Yew [58], Alexander J Trotter [70], Amy Trebes [98], George MacIntyre-Cockett [98], Alec Birchley [69], Alexander Adams [69], Amy Plimmer [69], Bree Gatica-Wilcox [69], Caoimhe McKerr [69], Ember Hilvers [69], Hannah Jones [69], Hibo Asad [69], Jason Coombes [69], Johnathan M Evans [69], Laia Fina [69], Lauren Gilbert [69], Lee Graham [69], Michelle Cronin [69], Sara Kumziene-SummerhaYes [69], Sarah Taylor [69], Sophie Jones [69], Danielle C Groves [93], Peijun Zhang [93], Marta Gallis [93] and Stavroula F Louka [93].

Samples and logistics, and Software and analysis tools:
Igor Starinskij [48].

Sequencing and analysis, and Software and analysis tools:
Chris J Illingworth [47], Chris Jackson [47], Marina Gourtovaia [99], Gerry Tonkin-Hill [99], Kevin Lewis [99], Jaime M Tovar-Corona [99], Keith James [99], Laura Baxter [94], Mohammad T. Alam [94], Richard J Orton [48], Joseph Hughes [48], Sreenu Vattipally [48], Manon Ragonnet-Cronin [39], Fabricia F. Nascimento [39], David Jorgensen [39], Olivia Boyd [39],

Lily Geidelberg [39], Alex E Zarebski [23], Jayna Raghwani [23], Moritz UG Kraemer [23], Joel Southgate [10, 69], Benjamin B Lindsey [93] and Timothy M Freeman [93].

Software and analysis tools, and Visualisation:
Jon-Paul Keatley [99], Joshua B Singer [48], Leonardo de Oliveira Martins [70], Corin A Yeats [14], Khalil Abudahab [14, 114], Ben EW Taylor [14, 114] and Mirko Menegazzo [14].

Leadership and supervision:
John Danesh [99], Wendy Hogsden [46], Sahar Eldirdiri [21], Anita Kenyon [21], Jenifer Mason [43], Trevor I Robinson [43], Alison Holmes [38, 103], James Price [38, 103], John A Hartley [82], Tanya Curran [3], Alison E Mather [70], Giri Shankar [69], Rachel Jones [69], Robin Howe [69] and Sian Morgan [9].

Metadata curation:
Elizabeth Wastenge [53], Michael R Chapman [34, 88, 99], Siddharth Mookerjee [38, 103], Rachael Stanley [54], Wendy Smith [15], Timothy Peto [59], David Eyre [59], Derrick Crook [59], Gabrielle Vernet [33], Christine Kitchen [10], Huw Gulliver [10], Ian Merrick [10], Martyn Guest [10], Robert Munn [10], Declan T Bradley [63, 72] and Tim Wyatt [63].

Project administration:
Charlotte Beaver [99], Luke Foulser [99], Sophie Palmer [88], Carol M Churcher [88], Ellena Brooks [88], Kim S Smith [88], Katerina Galai [88], Georgina M McManus [88], Frances Bolt [38, 103], Francesc Coll [19], Lizzie Meadows [70], Stephen W Attwood [23], Alisha Davies [69], Elen De Lacy [69], Fatima Downing [69], Sue Edwards [69], Garry P Scarlett [76], Sarah Jeremiah [83] and Nikki Smith [93].

Samples and logistics:
Danielle Leek [88], Sushmita Sridhar [88, 99], Sally Forrest [88], Claire Cormie [88], Harmeet K Gill [88], Joana Dias [88], Ellen E Higginson [88], Mailis Maes [88], Jamie Young [88], Michelle Wantoch [7], Sanger Covid Team (www.sanger.ac.uk/covid-team) [99], Dorota Jamrozy [99], Stephanie Lo [99], Minal Patel [99], Verity Hill [90], Claire M Bewshea [91], Sian Ellard [73, 91], Cressida Auckland [73], Ian Harrison [66], Chloe Bishop [66], Vicki Chalker [66], Alex Richter [85], Andrew Beggs [85], Angus Best [86], Benita Percival [86], Jeremy Mirza [86], Oliver Megram [86], Megan Mayhew [86], Liam Crawford [86], Fiona Ashcroft [86], Emma Moles-Garcia [86], Nicola Cumley [86], Richard Hopes [64], Patawee Asamaphan [48], Marc O Niebel [48], Rory N Gunson [100], Amanda Bradley [52], Alasdair Maclean [52], Guy Mollett [52], Rachel Blacow [52], Paul Bird [16], Thomas Helmer [16], Karlie Fallon [16], Julian Tang [16], Antony D Hale [49], Louissa R Macfarlane-Smith [49], Katherine L Harper [49], Holli Carden [49], Nicholas W Machin [45, 64], Kathryn A Jackson [92], Shazaad S Y Ahmad [45, 64], Ryan P George [45], Lance Turtle [92], Elaine O'Toole [43], Joanne Watts [43], Cassie Breen [43], Angela Cowell [43], Adela Alcolea-Medina [32, 96], Themoula Charalampous [12, 42], Amita Patel [11], Lisa J Levett [35], Judith Heaney [35], Aileen Rowan [39], Graham P Taylor [39], Divya Shah [30], Laura Atkinson [30], Jack CD Lee [30], Adam P Westhorpe [82], Riaz Jannoo [82], Helen L Lowe [82], Angeliki Karamani [82], Leah Ensell [82], Wendy Chatterton [35], Monika Pusok [35], Ashok Dadrah [75], Amanda Symmonds [75], Graciela Sluga [44], Zoltan Molnar [72], Paul Baker [79], Stephen Bonner [79], Sarah Essex [79], Edward Barton [56], Debra Padgett [56], Garren Scott [56], Jane Greenaway [57], Brendan AI Payne [50], Shirelle Burton-Fanning [50], Sheila Waugh [50], Veena Raviprakash [17], Nicola Sheriff [17], Victoria Blakey [17], Lesley-Anne Williams [17], Jonathan Moore [27], Susanne Stonehouse [27], Louise Smith [55], Rose K Davidson [89], Luke Bedford [26], Lindsay Coupland [54], Victoria Wright [18], Joseph G Chappell [97], Theocharis Tsoleridis [97], Jonathan Ball [97], Manjinder Khakh [15], Vicki M Fleming [15], Michelle M Lister [15], Hannah C Howson-Wells [15], Louise Berry [15], Tim Boswell [15], Amelia Joseph [15], Iona Willingham [15], Nichola Duckworth [60], Sarah Walsh [60], Emma Wise [2, 111], Nathan Moore [2, 111], Matilde Mori [2, 108, 111], Nick Cortes [2, 111], Stephen Kidd [2, 111], Rebecca Williams [33], Laura Gifford [69], Kelly Bicknell [61], Sarah Wyllie [61], Allyson Lloyd [61], Robert Impey [61], Cassandra S Malone [6], Benjamin J Cogger [6], Nick Levene [62], Lynn Monaghan [62], Alexander J Keeley [93], David G Partridge [78, 93], Mohammad Raza [78, 93], Cariad Evans [78, 93] and Kate Johnson [78, 93].

Sequencing and analysis:
Emma Betteridge [99], Ben W Farr [99], Scott Goodwin [99], Michael A Quail [99], Carol Scott [99], Lesley Shirley [99], Scott AJ Thurston [99], Diana Rajan [99], Iraad F Bronner [99], Louise Aigrain [99], Nicholas M Redshaw [99], Stefanie V Lensing [99], Shane McCarthy [99], Alex Makunin [99], Carlos E Balcazar [90], Michael D Gallagher [90], Kathleen A Williamson [90], Thomas D Stanton [90], Michelle L Michelsen [91], Joanna Warwick-Dugdale [91], Robin Manley [91], Audrey Farbos [91], James W Harrison [91], Christine M Sambles [91], David J Studholme [91], Angie Lackenby [66],

Tamyo Mbisa [66], Steven Platt [66], Shahjahan Miah [66], David Bibby [66], Carmen Manso [66], Jonathan Hubb [66], Gavin Dabrera [66], Mary Ramsay [66], Daniel Bradshaw [66], Ulf Schaefer [66], Natalie Groves [66], Eileen Gallagher [66], David Lee [66], David Williams [66], Nicholas Ellaby [66], Hassan Hartman [66], Nikos Manesis [66], Vineet Patel [66], Juan Ledesma [67], Katherine A Twohig [67], Elias Allara [64, 88], Clare Pearson [64, 88], Jeffrey K. J. Cheng [94], Hannah E. Bridgewater [94], Lucy R. Frost [94], Grace Taylor-Joyce [94], Paul E Brown [94], Lily Tong [48], Alice Broos [48], Daniel Mair [48], Jenna Nichols [48], Stephen N Carmichael [48], Katherine L Smollett [40], Kyriaki Nomikou [48], Elihu Aranday-Cortes [48], Natasha Johnson [48], Seema Nickbakhsh [48, 68], Edith E Vamos [92], Margaret Hughes [92], Lucille Rainbow [92], Richard Eccles [92], Charlotte Nelson [92], Mark Whitehead [92], Richard Gregory [92], Matthew Gemmell [92], Claudia Wierzbicki [92], Hermione J Webster [92], Chloe L Fisher [28], Adrian W Signell [20], Gilberto Betancor [20], Harry D Wilson [20], Gaia Nebbia [12], Flavia Flaviani [31], Alberto C Cerda [96], Tammy V Merrill [96], Rebekah E Wilson [96], Marius Cotic [82], Nadua Bayzid [82], Thomas Thompson [72], Erwan Acheson [72], Steven Rushton [51], Sarah O'Brien [51], David J Baker [70], Steven Rudder [70], Alp Aydin [70], Fei Sang [18], Johnny Debebe [18], Sarah Francois [23], Tetyana I Vasylyeva [23], Marina Escalera Zamudio [23], Bernardo Gutierrez [23], Angela Marchbank [10], Joshua Maksimovic [9], Karla Spellman [9], Kathryn McCluggage [9], Mari Morgan [69], Robert Beer [9], Safiah Afifi [9], Trudy Workman [10], William Fuller [10], Catherine Bresner [10], Adrienn Angyal [93], Luke R Green [93], Paul J Parsons [93], Rachel M Tucker [93], Rebecca Brown [93] and Max Whiteley [93].

Software and analysis tools:
James Bonfield [99], Christoph Puethe [99], Andrew Whitwham [99], Jennifier Liddle [99], Will Rowe [41], Igor Siveroni [39], Thanh Le-Viet [70] and Amy Gaskin [69].

Visualisation:
Rob Johnson [39].

1 Barking, Havering and Redbridge University Hospitals NHS Trust, **2** Basingstoke Hospital, **3** Belfast Health & Social Care Trust, **4** Betsi Cadwaladr University Health Board, **5** Big Data Institute, Nuffield Department of Medicine, University of Oxford, **6** Brighton and Sussex University Hospitals NHS Trust, **7** Cambridge Stem Cell Institute, University of Cambridge, **8** Cambridge University Hospitals NHS Foundation Trust, **9** Cardiff and Vale University Health Board, **10** Cardiff University, **11** Centre for Clinical Infection & Diagnostics Research, St. Thomas' Hospital and Kings College London, **12** Centre for Clinical Infection and Diagnostics Research, Department of Infectious Diseases, Guy's and St Thomas' NHS Foundation Trust, **13** Centre for Enzyme Innovation, University of Portsmouth (PORT), **14** Centre for Genomic Pathogen Surveillance, University of Oxford, **15** Clinical Microbiology Department, Queens Medical Centre, **16** Clinical Microbiology, University Hospitals of Leicester NHS Trust, **17** County Durham and Darlington NHS Foundation Trust, **18** Deep Seq, School of Life Sciences, Queens Medical Centre, University of Nottingham, **19** Department of Infection Biology, Faculty of Infectious & Tropical Diseases, London School of Hygiene & Tropical Medicine, **20** Department of Infectious Diseases, King's College London, **21** Department of Microbiology, Kettering General Hospital, **22** Departments of Infectious Diseases and Microbiology, Cambridge University Hospitals NHS Foundation Trust; Cambridge, UK, **23** Department of Zoology, University of Oxford, **24** Division of Virology, Department of Pathology, University of Cambridge, **25** East Kent Hospitals University NHS Foundation Trust, **26** East Suffolk and North Essex NHS Foundation Trust, **27** Gateshead Health NHS Foundation Trust, **28** Genomics Innovation Unit, Guy's and St. Thomas' NHS Foundation Trust, **29** Gloucestershire Hospitals NHS Foundation Trust, **30** Great Ormond Street Hospital for Children NHS Foundation Trust, **31** Guy's and St. Thomas' BRC, **32** Guy's and St. Thomas' Hospitals, **33** Hampshire Hospitals NHS Foundation Trust, **34** Health Data Research UK Cambridge, **35** Health Services Laboratories, **36** Heartlands Hospital, Birmingham, **37** Hub for Biotechnology in the Built Environment, Northumbria University, **38** Imperial College Hospitals NHS Trust, **39** Imperial College London, **40** Institute of Biodiversity, Animal Health & Comparative Medicine, **41** Institute of Microbiology and Infection, University of Birmingham, **42** King's College London, **43** Liverpool Clinical Laboratories, **44** Maidstone and Tunbridge Wells NHS Trust, **45** Manchester University NHS Foundation Trust, **46** Microbiology Department, Wye Valley NHS Trust, Hereford, **47** MRC Biostatistics Unit, University of Cambridge, **48** MRC-University of Glasgow Centre for Virus Research, **49** National Infection Service, PHE and Leeds Teaching Hospitals Trust, **50** Newcastle Hospitals NHS Foundation Trust, **51** Newcastle University, **52** NHS Greater Glasgow and Clyde, **53** NHS Lothian, **54** Norfolk and Norwich University Hospital, **55** Norfolk County Council, **56** North Cumbria Integrated Care NHS Foundation Trust, **57** North Tees and Hartlepool NHS Foundation Trust, **58** Northumbria University, **59** Oxford University Hospitals NHS Foundation Trust, **60** PathLinks, Northern Lincolnshire & Goole NHS Foundation Trust, **61** Portsmouth Hospitals University NHS Trust, **62** Princess Alexandra Hospital Microbiology Dept., **63** Public Health Agency, **64** Public Health England, **65** Public Health England, Clinical Microbiology and Public Health Laboratory, Cambridge, UK, **66** Public Health England, Colindale, **67** Public

Health England, Colindale, **68** Public Health Scotland, **69** Public Health Wales NHS Trust, **70** Quadram Institute Bioscience, **71** Queen Elizabeth Hospital, **72** Queen's University Belfast, **73** Royal Devon and Exeter NHS Foundation Trust, **74** Royal Free NHS Trust, **75** Sandwell and West Birmingham NHS Trust, **76** School of Biological Sciences, University of Portsmouth (PORT), **77** School of Pharmacy and Biomedical Sciences, University of Portsmouth (PORT), **78** Sheffield Teaching Hospitals, **79** South Tees Hospitals NHS Foundation Trust, **80** Swansea University, **81** University Hospitals Southampton NHS Foundation Trust, **82** University College London, **83** University Hospital Southampton NHS Foundation Trust, **84** University Hospitals Coventry and Warwickshire, **85** University of Birmingham, **86** University of Birmingham Turnkey Laboratory, **87** University of Brighton, **88** University of Cambridge, **89** University of East Anglia, **90** University of Edinburgh, **91** University of Exeter, **92** University of Liverpool, **93** University of Sheffield, **94** University of Warwick, **95** University of Cambridge, **96** Viapath, Guy's and St Thomas' NHS Foundation Trust, and King's College Hospital NHS Foundation Trust, **97** Virology, School of Life Sciences, Queens Medical Centre, University of Nottingham, **98** Wellcome Centre for Human Genetics, Nuffield Department of Medicine, University of Oxford, **99** Wellcome Sanger Institute, **100** West of Scotland Specialist Virology Centre, NHS Greater Glasgow and Clyde, **101** Department of Medicine, University of Cambridge, **102** Ministry of Health, Sri Lanka, **103** NIHR Health Protection Research Unit in HCAI and AMR, Imperial College London, **104** North West London Pathology, **105** NU-OMICS, Northumbria University, **106** University of Kent, **107** University of Oxford, **108** University of Southampton, **109** University of Southampton School of Health Sciences, **110** University of Southampton School of Medicine, **111** University of Surrey, **112** Warwick Medical School and Institute of Precision Diagnostics, Pathology, UHCW NHS Trust, **113** Wellcome Africa Health Research Institute Durban and **114** Wellcome Genome Campus.
